# A novel anti-virulence gene revealed by proteomic analysis in *Shigella flexneri *2a

**DOI:** 10.1186/1477-5956-8-30

**Published:** 2010-06-12

**Authors:** Ge Zhao, Li Zhu, Erling Feng, Xiaoyu Cao, Na Shang, Xiankai Liu, Xiang Liao, Tianyi Ying, Jie Wang, Huipeng Chen, Hengliang Wang

**Affiliations:** 1 Beijing Institute of Biotechnology, State Key Laboratory of Pathogen and Biosecurity, Beijing 100071, China; 2 Shandong Eye Institute, Qingdao 266071, China; 3 National Center of Biomedical Analysis, Beijing 100850, China

## Abstract

**Background:**

*Shigella flexneri *is a gram-negative, facultative pathogen that causes the majority of communicable bacterial dysenteries in developing countries. The virulence factors of *S. flexneri *have been shown to be produced at 37 degrees C but not at 30 degrees C. To discover potential, novel virulence-related proteins of *S. flexneri*, we performed differential in-gel electrophoresis (DIGE) analysis to measure changes in the expression profile that are induced by a temperature increase.

**Results:**

The ArgT protein was dramatically down-regulated at 37 degrees C. In contrast, the ArgT from the non-pathogenic *E. coli *did not show this differential expression as in *S. flexneri*, which suggested that *argT *might be a potential anti-virulence gene. Competitive invasion assays in HeLa cells and in BALB/c mice with *argT *mutants were performed, and the results indicated that the over-expression of ArgT_Y225D _would attenuate the virulence of *S. flexneri*. A comparative proteomic analysis was subsequently performed to investigate the effects of ArgT in *S. flexneri *at the molecular level. We show that HtrA is differentially expressed among different derivative strains.

**Conclusion:**

Gene *argT *is a novel anti-virulence gene that may interfere with the virulence of *S. flexneri *via the transport of specific amino acids or by affecting the expression of the virulence factor, HtrA.

## Background

*Shigella flexneri *is a gram-negative, facultative pathogen that is the leading cause of communicable bacterial dysenteries in developing countries. The virulence genes of *S. flexneri *are located on a large plasmid that was acquired by horizontal gene transfer. These genes encode a type III secretion system (TTSS) apparatus that enables *S. flexneri *to invade epithelial cells in the lower gut of humans [1].

The expression of virulence genes is induced under growth conditions similar to those found at the site of invasion. For example, a temperature of 37°C is a particularly important environmental signal. Maurelli *et al*. [2] found that *S. flexneri *cultivated at 37°C produced kerato-conjunctivitis in guinea pigs and were able to penetrate and replicate in intestinal epithelial cells, but that bacteria grown at 30°C were phenotypically avirulent and noninvasive. Other studies in *S. flexneri *have focused on regulation of the expression of these virulence genes at different temperatures [1,3]. However, we expected that, in addition to known virulence genes, a large number of other genes would be differentially expressed in response to a change in environmental temperature. To obtain a global view of the expression-level response of *S. flexneri *induced by a temperature increase and to find potentially novel virulence-related proteins, we performed a comparative proteomic analysis using the differential in-gel electrophoresis (DIGE) technique and mass spectrometry. Interestingly, the abundance of protein ArgT was dramatically decreased at 37°C.

The evolution of bacterial pathogens from nonpathogenic ancestors is marked principally by the acquisition of virulence gene clusters on plasmids and pathogenicity islands via horizontal gene transfer [4]. Genes that are no longer compatible with the novel lifestyle of the pathogen are selectively inactivated either by point mutation, insertion, or deletion. These incompatible genes are defined as anti-virulence genes [5]. At present, several anti-virulence genes, such as *cadA*, *ompT*, *nadA *and *nadB*, have been identified in *S. flexneri *[5,6]. In our previous report [7], we have showed that protein ArgT is degraded by HtrA protease in *S. flexneri *grown at 37°C. This finding is validated by this paper using a different proteomic method (DIGE). ArgT, a lysine/arginine/ornithine-binding protein (LAO), localizes in the periplasmic space and is responsible for binding the amino acid substrates during the process of amino-acid transport into the cytoplasm in *E. coli *[8,9]. In *S. flexneri*, the function of ArgT has not been elucidated. Given that ArgT expression is dramatically down-regulated at 37°C and *S. flexneri *is phenotypically noninvasive at 30°C, we presume that the selective expression of *argT *in *S. flexneri *may be related to its virulence. In this study, we confirmed that *argT *functions as a novel anti-virulence gene by a competitive invasion assay using *argT *mutations and investigated its mechanism by comparative proteomic analysis.

## Materials and methods

### Bacterial strains and growth conditions

Strain 2457T (our lab) was the wild-type strain of *S. flexneri *2a used throughout this study. There is not much difference in the growth curve of *S. flexneri *2a 2457T grown at 30°C and 37°C. But the growth speed cultured at 37°C is more rapid than at 30°C. Both the *S. flexneri *2a 2457T cultured at 30°C and 37°C grew into early stationary phase with the same OD_600 _= 2.4. Strain *E. coli *DH5α (our lab) was used for gene cloning, and *E. coli *BL21 (DE3) (Qiagen, Germany) was used for protein expression. *E. coli *and *S. flexneri *strains were grown in LB. When necessary, antibiotics were added at the following concentrations: 50 μg of kanamycin/mL or 100 μg of ampicillin/mL.

### Construction of the plasmids and strains

The *argT *genes of *S. flexneri *and *E. coli *were amplified by PCR using primers a1 and a2 (Additional file 1, Table S1) from the 2457T and DH5α genomes, respectively. The PCR products were cloned into the pProEX-HTb vector (Invitrogen, USA, Ap^r^) using *BamH *I and *EcoR *I sites, generating the pProEX-HTb-argT^2457T ^(ArgT) and pProEX-HTb-argT^DH5α ^(ArgT^DH5α^) plasmids. Similarly, *argT *without a signal peptide was amplified with primers a3 and a2 and ligated into pGEX-6p-2, generating pGEX-6p-2-argT2^2457T ^(pGEX-argT2). The *argT *products that included the regulatory region were amplified with primers argTH1 and argTH2 and cloned into the pAK plasmid [constructed in our lab, using the *ori *from pACYC184 and *kan *from pKD4 [10], km^r^] using *BamH *I and *Sal *I sites, generating pAK-argT. To introduce site mutations into *argT*, PCR was performed with primers containing the desired mutations using the QuikChange^® ^Site-Directed Mutagenesis Kit (Stratagene, USA), according to the manufacturer's protocol. The desired site-mutated plasmids ArgT_A16T, _ArgT_L38P_, ArgT_Y225D _and pAK-argT_Y225D _were confirmed by sequencing.

The *argT *deletion mutant of *S. flexneri *(2457T*ΔargT*) was constructed by a modified form of *lambda red *recombination, as described previously [10]. Briefly, 500 bp upstream (a5) and 500 bp downstream (a3) of *argT *were independently amplified with the primer pairs a5p1/a5p2, and a3p1/a3p2. The products were combined into pET22b (Novagen, Germany, Ap^r^) with *kan *gene. 2457T carrying pKD46 [10] containing the *red *recombinase genes were grown at 30°C in the presence of 10 mM arabinose in order to induce the recombinase genes and then transformed with the gel-purified a5-Kan-a3 PCR products amplified with a5p1 and a3p2 primers. Recombinants were selected from LB plates containing kanamycin. The *argT *rescued strain was constructed by transformation of the pAK-argT plasmid into the *argT *deletion strain without the *kan *gene.

### Invasion assays

To test the virulence alterations in the mutant strains, a competition assay was carried out using a HeLa cells infection model [11,12] or a murine intranasal infection model [13,14] with some modifications. Each assay was repeated three times.

2457T/pAK-argT_Y225D_, as well 2457T/pAK and 2457T were grown to the early stationary phase, recovered at the same OD values and suspended in normal saline. Cell density was determined by plating. 2457T/pAK-argT_Y225D _and 2457T (or 2457T/pAK and 2457T) were mixed at a 1:1 (v/v) ratio, and about 100 MOI of bacteria were allowed to infect HeLa cells in antibiotic-free DMEM at 37°C for 4 hrs, with three repeats for each group. The cells were then washed with normal saline and fresh DMEM containing gentamicin (100 ng/mL) was added. After cultivation for another 24 hrs, the infected cells were lysed by the addition of 0.1% deoxycholate sodium to liberate the intracellular bacteria. Dilutions of the lysates were plated on LB plates and cultivated at 37°C. The next day, colonies were randomly selected from LB plates of each repeat and used to inoculate LB plates with 50 μg of kanamycin/mL. These plates were cultivated at 37°C overnight. The number of mutant strains and 2457T were then counted. The competitive index was calculated by the method of Camilli *et al*. [15] according to the formula (mutant number after invasion / wild-type strain number after invasion) / (mutant number before invasion / wild-type strain number before invasion), and student *t*-test was employed to determine the p-value. The invasion assays for the *argT *deletion mutant 2457T*ΔargT *were performed by the same method.

For the murine intranasal infection, about 10^6 ^CFU of mixed bacteria were administered in a total 20 μL saline split into the nares of 7 BALB/c mice. After 24 hrs, the bacteria recovered from the lungs of the mice were treated by the method described above, and the competitive index of each mutant was calculated.

### Comparative proteomic analysis

The preparation of whole-cell protein extracts from different 2457T strains cultured at 37°C or 30°C was performed as described previously [16]. The protein concentration of each sample was measured using the PlusOne 2-D Quant Kit (GE Healthcare, USA). The samples with 800 μg proteins were then treated with a 2-D Clean-Up Kit (GE Healthcare) and suspended in 350 μL rehydration buffer (7 M Urea, 2 M Thiourea, 4% CHAPS, 50 mM DTT, 0.5% IPG Buffer). IEF was performed by using pH 4-7 and pH 6-11 IPG strips (18 cm, GE Healthcare) to obtain better separation and more spots at 20°C for 60,000 V·h. After IEF, each strip was equilibrated. For the second dimension, vertical slab SDS-PAGE (12.5%) was performed for about 4.5 hrs at 30 mA/gel by using a Bio-Rad Protean II Xi apparatus (Bio-Rad, Hercules, USA). A biological replicate at least 3 times involved in the gels running. When using the DIGE (differential in-gel electrophoresis) technique, 50 μg proteins from each temperature were covalently labeled with a fluorescent dye, either cy5 or cy3 (GE Healthcare), mixed equally, and then separated by 2-DE. The image gel was scanned using a 633-nm laser for cy5 or a 543-nm laser for cy3. The entire DIGE process was protected from light. Image analysis was processed by DeCyder-DIA software for the DIGE profiles and ImageMaster 2D Platinum software for common 2-DE profiles (GE Healthcare).

The protein spots of interest were cut out, and in-gel protein digestion was performed as described previously [16]. Peptides from digested proteins were resolubilized in 2 μL of 0.5% triflouroacetic acid (TFA). The MALDI-TOF-MS measurement was performed on a Bruker Reflex III MALDI-TOF-MS (Bruker Daltonics, Germany) operating in reflectron mode with a 20 kV accelerating voltage and a 23 kV reflecting voltage. A saturated solution of α-cyano-4-hydroxycinnamic acid in 50% acetonitrile and 0.1% trifluoroactic acid was used as the matrix. One microliter of a 1:1 mixture of the matrix solution and sample solution was loaded into the Score384 target well. Mass accuracy for peptide mass fingerprint (PMF) analysis was calibrated using a 0.1 Da-0.2 Da external standard, and internal calibration was carried out with enzyme autolysis peaks at a resolution of 12,000. PMFs were identified using the Mascot program (Matrix Science Ltd, UK) by searching a proprietary *S. flexneri *2a 2457T database, and the results were checked against the NCBInr public database. Monoisotopic masses were used to search the databases, allowing a peptide mass accuracy of 0.3 Da and one partial cleavage. Oxidation of methionine and carbamidomethyl modification of cysteine was considered. For unambiguous identification of proteins by MALDI-TOF data, the constraint of a match of five or more peptides was applied.

### Protein purification and antibody production

The expression of fusion proteins was induced by adding 1 mM IPTG when cultures were grown to an OD_600 _of 0.6-0.9 at 30°C, after which they were continuously cultured for another 16 hrs at 20°C. The induced ArgT fusion protein was purified from the cultures using Glutathione Sepharose 4B beads (Amersham, UK). The purified GST-ArgT proteins were injected subcutaneously into each of two BALB/c female mice to generate polyclonal ArgT antibody. Sera from the immunized mice were collected and purified by affinity chromatography, according to the manufacturer's instructions (Pierce, USA).

### SDS-PAGE and Western-blot analysis

Samples were boiled and loaded onto 12% SDS-PAGE gels [17]. After electrophoresis, proteins were transferred to PVDF membranes (Immobilon-Millipore, USA) and blocked with 5% nonfat milk. Both incubations with primary antibody diluted 1:5000 and secondary antibody conjugated to horseradish peroxidase (Santa Cruz Biotechnology, USA) diluted 1:10000 in blocking buffer were performed for 1 h at room temperature. Immunoblots were washed three times with Tris-buffered saline containing 0.05% Tween 20 after both antibody incubations. The ECL Plus kit (Pierce) was used for detection.

## Results

### ArgT expression down-regulated at 37°C by DIGE analysis

**Figure 1:**
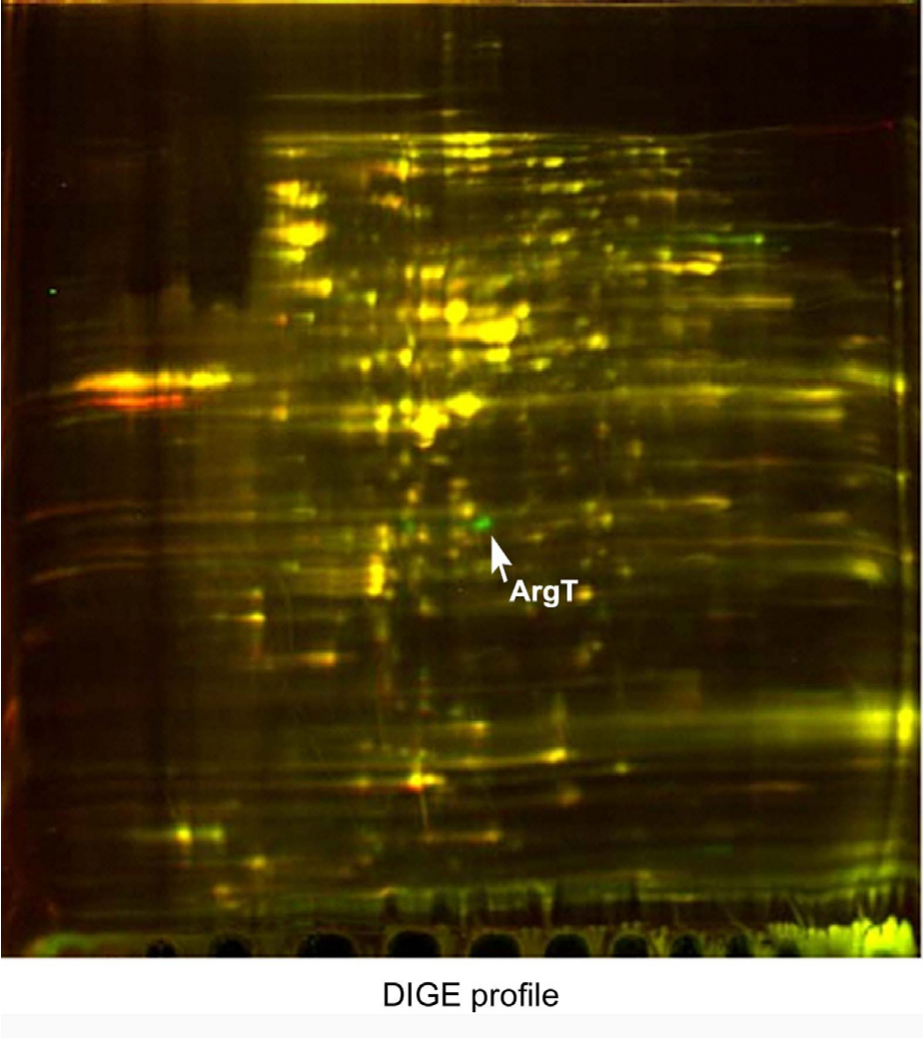
**DIGE profile of *S. flexneri***. The green spots represent proteins up-regulated at 30°C, the red spots represent proteins up-regulated at 37°C, and the yellow spots represent proteins without obvious differences between the two temperature conditions.

The DIGE profiles of 2457T grown to early stationary growth phase at 30°C and 37°C showed the proteins expressed at the two temperatures were very similar. A total of 922 spots were detected in the DIGE profile. A protein was considered to be temperature-dependent if the spot density was significantly different (two-fold or greater) under the two temperature conditions. Each differentially expressed spot was selected for identification by MALDI-TOF-MS. Among these differential spots, the abundance of ArgT was dramatically decreased at 37°C (Fig. 1), which was in contrast to the general virulence factors whose abundances were increased at 37°C. The expression amount of ArgT at 30°C is about 27 fold more than that at 37°C. However, The quantities of argT mRNA under the two temperatures were similar according to the results of real-time PCR (the mRNA abundance in 30°C is about 2^0.58 ^= 1.49 fold more than that in 37°C)(Data not shown). Based upon this observation, we next wanted to determine if ArgT was related to the virulence of *S. flexneri*.

### ArgT expression rescued by a single amino-acid residue site mutation in *S. flexneri argT*

**Figure 2:**
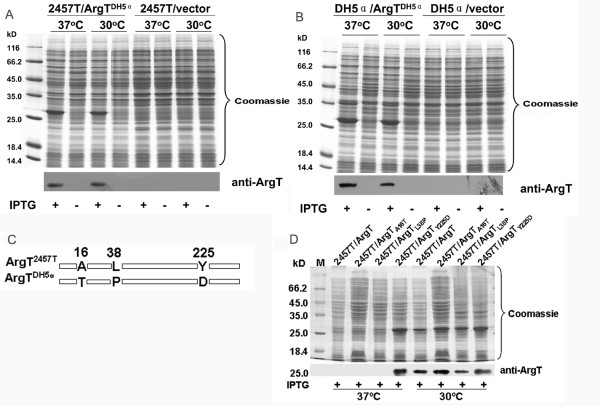
**Identification of the key site of 2457T ArgT (tyrosine residue in position 225) for the regulation of expression**. ArgT^DH5α ^over-expressed in 2457T (A) and in DH5α (B) could be detected either at 37°C or at 30°C. (C) The three corresponding different sites between the protein sequence of ArgT^2457T ^and ArgT^DH5α^. (D) Comparison of ArgT over-expressed in four strains carrying different plasmids at 37°C and 30°C. The plasmids include pProEX-HTb-argT^2457T ^(ArgT) and its site-mutated plasmids ArgT_A16T_, ArgT_L38P _and ArgT_Y225D_. M: protein marker (Fermentas, UK).

Protein ArgT of 2457T had been demonstrated to be a periplasmic protein in our previous work [7]. If the significantly differential expression of ArgT was related to the virulence of *S. flexneri*, this phenomenon should not occur in its non-pathogenic ancestor (*E. coli*). Thus, the *argT *gene from *E. coli *DH5α was cloned and over-expressed in both 2457T and DH5α. Regardless of the incubation temperature, the expression of *E. coli *ArgT can be detected in DH5α and 2457T (Fig. 2A). The different fates of *S. flexneri *and *E. coli *ArgT suggested that the sequence of the *S. flexneri argT *gene may have changed, endowing a survival advantage over its non-pathogenic ancestor during the long evolutionary process.

The expression product of *argT *in *S. flexneri *shares a 98.85% identity with its counterpart in *E. coli*. Comparison of the ArgT sequences of 2457T and DH5α revealed that there were only three amino acid changes, which were A16T, L38P and Y225D (Fig. 2B). These three amino acids in the *S. flexneri *ArgT were site-mutated into the corresponding *E. coli *ArgT amino acids to generate three different expression vectors (ArgT_A16T_, ArgT_L38P _and ArgT_Y225D_). The expression of ArgT in 2457T carrying ArgT_A16T _or ArgT_L38P _did not differ from that of the wild type, which indicated that the mutation of Ala into Thr or of Leu into Pro at these sites had no effect on the expression of ArgT. In contrast, the mutation of Tyr255 into Asp could rescue the expression of ArgT at 37°C (Fig. 2C). Since ArgT regulation has been demonstrated to be a posttranslational proteolysis event [7], the Tyr at the 225 position might be a key amino acid residue that is involved in the regulation, whose change renders the protein less susceptible to proteolytic attack.

### The virulence of 2457T attenuated by ArgT over-expression

**Figure 3:**
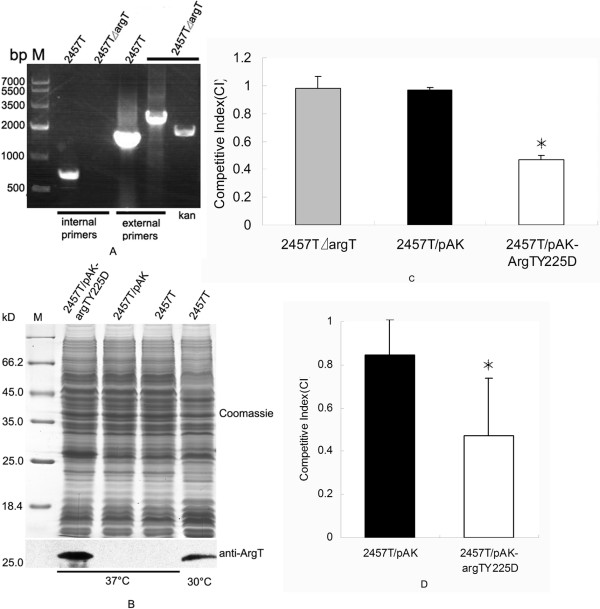
**Identification of the *argT *mutants and the results of invasion assays**. (A) Identification of the *argT *deletion mutant. (B) Identification of the ArgT over-expression strain. The confirmed *argT *mutant strains and the wild-type strain were used for competitive invasion assays in HeLa cells (C) and BALB/c mice (D), respectively. The results show that the competitive index of the *argT *deletion mutant in the cell invasion assay was the same as the control, while the competitive index of the ArgT over-expression strain in the two kinds of invasion assays were both significantly lower (*, P < 0.05) than the control.

To test the virulence-related function of *argT*, *argT *deletion and over-expression strains were constructed and used for an invasion assay. The *argT *deletion mutant was confirmed by PCR with the internal primers, external primers and *kan *primers (Fig. 3A), and the ArgT over-expression strain was detected by SDS-PAGE and Western blotting (Fig. 3B). Here, a recombinant strain carrying the low-copy expression vector pAK-argT_Y225D _(containing the *argT *of 2457T with the native regulatory region but mutated at position 225) was used as the ArgT over-expression strain to better simulate the state of the *argT *gene in vivo. The competitive invasion assays in HeLa cells and BALB/c mice by *argT *deletion and over-expression strains were performed in triplicate. Since the virulence could not be changed by *argT *deletion in our initial assay, the *argT *deletion strain was not used in the later mice invasion assays. The competitive index (CI) was calculated and is shown in Figs. 3C and 3D. From these data, we concluded that *argT *deletion had no impact on the virulence of 2457T; however, ArgT over-expression attenuated the virulence at 37°C.

### Differentially expressed proteins in the comparative proteomic analysis

**Figure 4:**
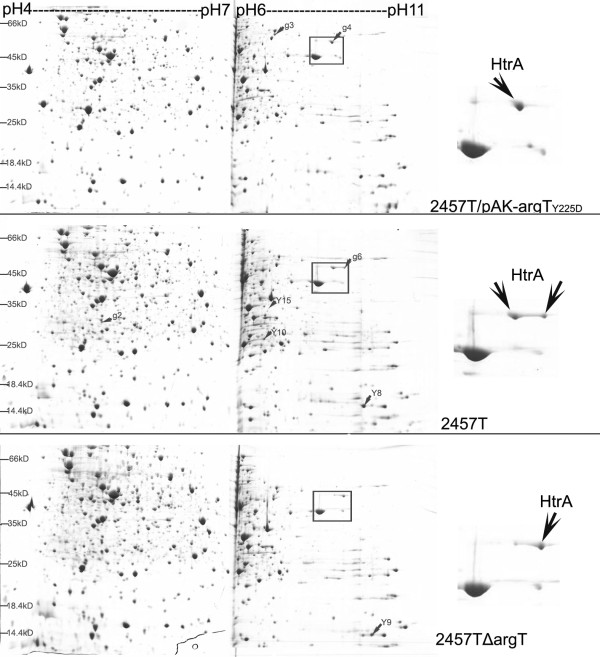
**Protein expression profiles of different ArgT expression strains at 37°C**. Proteins were separated by IEF in the first dimension (18 cm) with the p*I *range of pH 4-7 and pH6-11 and by 12.5% SDS-PAGE in the second dimension and then stained by Coomassie. HtrA presents two spots in the expression profile of 2457T. The abundance of the left is higher than the right in the wild type, while the abundance of the right is higher than the left in *argT *deletion mutant. Only the left can be detected in the ArgT over-expression strain.

In addition to confirming the above hypothesis that ArgT depresses the virulence of *S. flexneri *by invasion assay, we also searched for clues of the mechanism at the molecular level by comparative proteomic analysis. The protein expression profiles of wild-type 2457T, the *argT *deletion mutant and the ArgT over-expression strain at 37°C are shown in Fig. 4. The 2-DE profiles of different *argT *mutants were compared with the wild-type 2457T, and they were highly comparable. Only eight spots with expression level differences greater than two-fold were excised for further analysis (Additional file 1, Table S2). Since there is no obvious difference in growth curve and biochemical reactions tested by API strips (data not shown) between the wild strain and its derivative strains, we concluded that the change in protein expression was not caused by manipulation.

The differential protein expression between the *argT *mutant and wild-type strains might be related to the function of ArgT. Among the differentially expressed proteins, HtrA attracted our attention because of its potential function as a virulence factor [18]. The expression of HtrA varied with the different expression levels of ArgT. Two spots were identified as HtrA in the wild-type strains, and the abundance of the left was higher than the right (Fig. 4). When *argT *was deleted, however, the abundance of the right spot was higher than the left. Only the left spot was detectable in the ArgT over-expression strain. However, separation of large proteins at high pI regions is more susceptible. We will do further study to verify the reliability of this phenomenon in the next work.

## Discussion

Comparative analysis of virulence potential is a cornerstone in understanding the ability of a pathogen to cause disease and in determining the relative contribution of putative virulence factors. Using this method, we found that ArgT over-expression could attenuate the virulence at 37°C. This result was consistent with that of the comparative proteomic analysis of 2457T at 37°C and 30°C, in which ArgT was normally expressed when 2457T was noninvasive but not required when 2457T was invasive. In addition, we showed that 2457T ArgT disappeared at 37°C, while DH5α ArgT could be detected at 37°C. These observations are in line with the criteria for the identification of anti-virulence genes that were proposed by Dr. Maurelli: 1) anti-virulence genes are present and expressed in *E. coli *but deleted or mutated in *S. flexneri*; and 2) their compulsive expression in *S. flexneri *can attenuate virulence [5].

Bacterial pathogens can either acquire virulence genes or lose anti-virulence genes through horizontal gene transfer during their evolution, and examination of the latter has just begun in recent years [5]. Selective pressure can lead to the deletion or inactivation of genes that are no longer compatible with the lifestyle of the pathogen. A few examples include *cadA*, *ompT*, *nadA *and *nadB *[5,6], which are all absent or inactive in *S. flexneri *but present and expressed in the non-virulent ancestral *E. coli*. The expression of any of these genes in the pathogen would be adverse to the expression of virulence genes [5]. In this case, *argT*, which has been identified as a novel anti-virulence gene, is not totally absent in *S. flexneri*. Instead, it is expressed at 30°C. One potential explanation could be that its role in amino acid transport facilitates the survival of *S. flexneri *in non-host environments and, thus, provides a selective advantage for those cells that harbor this gene.

Although ArgT may be a novel anti-virulence gene, the mechanism by which ArgT interferes with the invasion of *S. flexneri *remains unknown. One possibility could be that the amino acids that are transported by ArgT depress the virulence. For example, lysine could be decarboxylated to cadaverine by lysine decarboxylase, and the latter could block the ability of *S. flexneri *to elicit transepithelial migration of polymorphonuclear neutrophils for the inflammatory response [19]. If this possibility exists, there must be a new virulence inhibitor other than cadaverine, because the gene encoding lysine decarboxylase (*cadA*) has been deleted in the genome of *S. flexneri*. In addition, ornithine, in conjunction with uracil, has been reported to significantly reduce the hemolytic ability of wild-type cultures and the expression of the type III secretion system [20]. Another possibility could be related to HtrA, whose expression was varied with the different expression levels of ArgT. HtrA is a periplasmic protease that participates in the processing or modification of surface proteins and the degradation of abnormal or incorrectly folded proteins [21]. Here, however, we were more interested in the virulence-related function of HtrA. HtrA has been reported to facilitate the surface expression of IcsA and is required for the efficient intercellular spread of *S. flexneri *[22]. HtrA has also been found to be a virulence factor in other bacteria, such as *Salmonella typhimurium *[23] and *Listeria monocytogenes *[24]. In this study, the two spots (left and right) of HtrA in the proteomic profile might correspond to two different modified forms and the right form might be related to virulence due to its presence in strains with invasive ability. Since ArgT of 2457T has been demonstrated to be located in periplasm [7], and HtrA is also a periplasmic protein in *S. flexneri*, the two proteins must interact with each other directly or indirectly. We suggested that, if ArgT could not be degraded by HtrA, as ArgT_Y225D _did, it might influence the modification of HtrA, which would subsequently influence the virulence of *S. flexneri*. The putative mechanism by which this modification occurs and interferes with virulence will require further study.

Besides HtrA, some other proteins such as OmpA and PepA showed differential expression in the derived 2457T strains. OmpA is an outer membrane protein, which can function as an adhesion and invasion, participate in biofilm formation, act as both an immune target and evasion, and serves as a receptor for several bacteriophages [25]. Here, OmpA is down-regulated expression in ArgT over-expression 2457T strains, and the virulence of this strain is decreased, these phenomena just manifested that OmpA is a potential virulence factor in 2457T due to its adhesion and invasion functions. PepA is a leucyl aminopeptidase, and no reports mentioned that PepA was involved in virulence, so here its up-regulated expression in ArgT over-expression 2457T strains may be related to the amino acid metabolism due to the amino-acid transportation function of ArgT.

Anti-virulence genes provide us with new insights into the ecology and evolution of bacterial pathogens. The study of anti-virulence genes holds the potential for discovery of new pathogen-specific therapies and the development of safer, attenuated vaccine strains. How could we find anti-virulence genes? Traditional methods that focus on laboratory phenotypes have been proven successful but laborious. The comparative genomic strategies that exploit data provided by genome sequencing studies are well accepted for the identification of anti-virulence genes. However, those anti-virulence genes still present in the genome of pathogens like *argT *can not be identified through genomic analysis. Comparative proteomics is one of the best supplemental approaches. Since proteins are the functional output of genes, analysis of the changes in proteome of pathogens grown at conditions similar to their host environments will uncover those precisely regulated and selectively expressed genes. So, it's reasonable that comparative proteomics will eventually become another powerful tool to discover additional anti-virulence genes in this post-genome era.

## Conclusion

ArgT functions as an anti-virulence factor that potentially interferes with the invasion ability of *S. flexneri *either through the transportation of specific amino acids or by an influence on the expression of the virulence factor HtrA.

## Abbreviations

DIGE: differential in-gel electrophoresis; TTSS: type III secretion system; LAO: lysine/arginine/ornithine-binding protein; 2-DE: Two-dimensional Polyacrylamide Gel Electrophoresis; MALDI-TOF-MS: matrix-assisted laser desorption ionisation- time of flight-mass spectrometry; PMF: peptide mass fingerprint; CI: competitive index.

## Competing interests

The authors declare that they have no competing interests.

## Authors' contributions

GZ and XYC performed the mutants construction and invasion assay experiments. LZ, XL and TYY carried out the 2-D PAGE experiments. ELF and XKL conducted the database search and bioinformatics analyses. JW operated the MALDI-TOF MS instrument and calibrated all of the original MS data. NS performed Western Blotting analysis. GZ, LZ and HLW were responsible for data analysis and drafting the manuscript. HPC and HLW participated in the study design and helped to draft the manuscript. All authors read and approved the final manuscript.

## Supplementary Material

Additional file 1 **Table S1 - Primers used in this study and Table S2**. Identification of 8 differential-expression proteins by MALDI-TOF MS between *argT *mutants and wild-type strain. Click here for file
