# Supplementary material for: A novel anti-virulence gene revealed by proteomic analysis in Shigella flexneri 2a
Source: Proteome Sci. 2010 Jun 12;8:30. doi: 10.1186/1477-5956-8-30 (PMC2904734; doi:10.1186/1477-5956-8-30)
Supplement: Additional file 1 — Table S1 - Primers used in this study and Table S2. Identification of 8 differential-expression proteins by MALDI-TOF MS between argT mutants and wild-type strain. [file 1477-5956-8-30-S1.DOC]

Table S1. Primers used in this study.

| primer | sequence |
| --- | --- |
| primer a1 | 5′-GACTGGATCCATGAAGAAGTCGATTCTCG-3′ |
| primer a3 | 5′-TACGGGATCCGCGCTACCGGAGACGGTACGTAT-3′ |
| primer a2 | 5′-CTGAGAATTCTCAGTCACCGTAGACATTAAAG-3′ |
| argTH1 | 5′-ACTGGGATTCCTGTTGCCCTGTTTCAGG-3′ |
| argTH2 | 5′-GCGTCGACGTTTGTTACACTTATCGTGCC-3′ |
| A16T1 | 5′-TTAGTCGGTCTCTCCACAGCGGCTTCCAG-3′ |
| A16T2 | 5′-CTGGAAGCCGCTGTGGAGAGACCGACTAA-3′ |
| P38L1 | 5′-GATACCACCTATGCACTGTTCTCGTCGAAAG-3′ |
| P38L2 | 5′-CTTTCGACGAGAACAGTGCATAGGTGGTATC-3′ |
| Y225D1 | 5′-GCTACGTAAAGATGATGCTGAACTGACG-3′ |
| Y225D2 | 5′-CGTCAGTTCAGCATCATCTTTACGTAGC-3′ |
| kanp1 | 5′-GCGTCGACGTGTAGGCTGGAGCTGCTTC-3′ |
| kanp2 | 5′-CCAAGCTTATGGGAATTAGCCATGGTCC-3′ |
| a5p1 | 5′-CGGGATCCGGCAATTTCTTGCTGACG-3′ |
| a5p2 | 5′-GCGTCGACCTGGGAAGCTGTACCTGATG-3′ |
| a3p1 | 5′-CCAAGCTTGTCTTATGCCATCTTGACG-3′ |
| a3p2 | 5′-CCCTCGAGCTCTTCCGGTTCTTTCCA-3′ |
| argTU | 5′-TCTGTCTTTATTAGTCGGTCTC-3′ |
| argTL | 5′-AGCCGTCAGTTCAGCATA-3′ |

Table S2. Identification of 8 differential-expression proteins by MALDI-TOF MS between *argT* mutants and wild-type strain.

| *Spot NO.^a^* | *Protein symbol* | *GI NO.* | *Score* | *Mactched/ Searched* | *MW* | *PI* | *Expression mode^b^* | *Fold difference* | *Localization^c^* | **Protein description** |
| --- | --- | --- | --- | --- | --- | --- | --- | --- | --- | --- |
| Y8 | RplJ | 30064732 | 83 | 13/74 | 17757 | 9.04 | **↓** | 0.375 | U | 50S ribosomal protein L10 |
| Y9 | RplJ | 30064732 | 106 | 13/43 | 17757 | 9.04 | **↑** | 4.679 | U | 50S ribosomal protein L10 |
| Y10 | NfnB | 30062032 | 117 | 11/39 | 23936 | 6.38 | **↓** | 0.212 | U | oxygen-insensitive NAD(P)H nitroreductase |
| Y15 | HybA | 30064351 | 72 | 8/47 | 36949 | 7.1 | **↓** | 0.466 | P | hydrogenase-2 small subunit |
| g2 | OmpA | 30062494 | 107 | 13/50 | 37374 | 5.65 | **↓** | 0.236 | OM | outer membrane protein 3a |
| g3 | PepA | 30065453 | 175 | 30/85 | 55403 | 6.82 | **↑** | 2.421 | C | leucyl aminopeptidase |
| g4 | HtrA | 30061721 | 134 | 18/42 | 49465 | 8.84 | **↑** | 9.267 | P | periplasmic serine protease Do, heat shock protein |
| **g6** | HtrA | 30061721 | 144 | 14/30 | 49351 | 8.84 | **↓** | 0.108 | P | periplasmic serine protease Do, heat shock protein |

^a^ Y representing argT deletion mutant; g representing ArgT over-expressed mutant.

^b^↑ representing up-regulation in mutant compared to wild-type strain; ↓ representing down-regulation in mutant compared to wild-type strain.

^c^ Protein localization was predicted by PSORTb v2.0. U: unknown, P: periplasm, OM: outer membrane, C: cytoplasm.
